# PM2.5 Exposure and Asthma Development: The Key Role of Oxidative Stress

**DOI:** 10.1155/2022/3618806

**Published:** 2022-04-04

**Authors:** Kaimeng Liu, Shucheng Hua, Lei Song

**Affiliations:** Department of Respiratory Medicine, Center for Pathogen Biology and Infectious Diseases, Key Laboratory of Organ Regeneration and Transplantation of the Ministry of Education, State Key Laboratory for Zoonotic Diseases, The First Hospital of Jilin University, Changchun 130021, China

## Abstract

Oxidative stress is defined as the imbalance between reactive oxygen species (ROS) production and the endogenous antioxidant defense system, leading to cellular damage. Asthma is a common chronic inflammatory airway disease. The presence of asthma tends to increase the production of reactive oxygen species (ROS), and the antioxidant system in the lungs is insufficient to mitigate it. Therefore, asthma can lead to an exacerbation of airway hyperresponsiveness and airway inflammation. PM2.5 exposure increases ROS levels. Meanwhile, the accumulation of ROS will further enhance the oxidative stress response, resulting in DNA, protein, lipid, and other cellular and molecular damage, leading to respiratory diseases. An in-depth study on the relationship between oxidative stress and PM2.5-related asthma is helpful to understand the pathogenesis and progression of the disease and provides a new direction for the treatment of the disease. This paper reviews the research progress of oxidative stress in PM2.5-induced asthma as well as highlights the therapeutic potentials of antioxidant approaches in treatment of asthma.

## 1. Introduction

Asthma is a common chronic inflammatory airway disease that affected an estimated 358 million people in 2015, and it can affect both children and adults [1]. Although the prevalence, severity, and mortality of asthma vary globally, it remains one of the most common chronic diseases that cause significant morbidity and mortality [2], which has posed a serious threat to human health and economic and social development. Even in America, asthma-related exacerbations are reported to result in about 2 million emergency department (ED) visits and 500,000 hospitalizations each year [3].

Asthma is a complex and chronic inflammatory disease of the airways with heterogeneity in etiology, pathogenesis, clinical manifestations, and prognosis. Airway inflammation, airway hyperresponsiveness, and airway remodeling are recognized as the central pathophysiological features of asthma. In recent years, oxidative stress in asthma has drawn more and more attention due to the increasing and ever-growing environmental concerns. Environmental factors such as pollutants and (non)ionizing radiation can produce many ROS, resulting in oxidative stress [4]. Oxidative stress may attack lipids, proteins, and DNA, with deleterious consequences for respiratory diseases. Furthermore, many oxygen free radicals are produced during inflammatory cell recruitment and activation in asthma, thus triggering lipid peroxidation and causing inflammatory responses as well as tissue damage [5].

In recent years, epidemiological studies have strongly suggested that an increased risk of asthma exacerbations is associated with elevated exposure to air pollution, especially PM2.5 exposure [6]. Substantial epidemiological investigations have revealed that exposure to PM2.5 is closely correlated to the progression of numerous respiratory diseases, leading to airway inflammation, a decline in lung function, and exacerbation and progression of chronic obstructive pulmonary disease (COPD) and asthma [7–11]. Many experiments have proved that PM2.5 exposure contributes to the increased risk of respiratory infections [12–16]. Furthermore, PM2.5 exposure has long been associated with increased morbidity and mortality from lung diseases such as COPD and asthma [6, 17]. Numerous studies have shown that PM2.5 exposure could produce excessive ROS and thus reduce antioxidant enzyme activities, which results in oxidative stress in cells [18, 19].

PM2.5 exposure can significantly increase the level of oxidative stress, which has an important role in the development of asthma. This paper expounds on the effects of oxidative stress on PM2.5 and asthma, demonstrating how oxidative stress affects airway inflammation, airway hyperresponsiveness, and airway remodeling in the development of asthma under PM2.5 exposure.

## 2. The Presence of Oxidative Stress in Asthma

**Figure 1:**
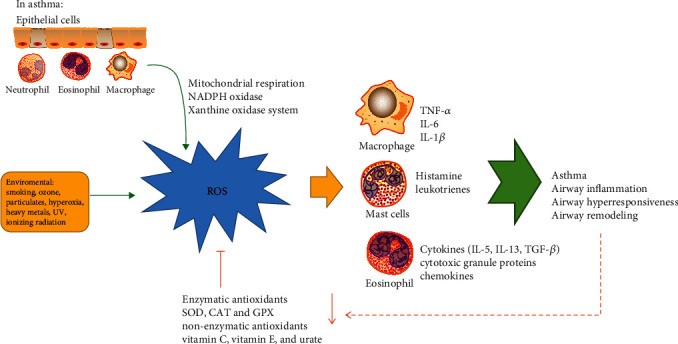
The oxidative and antioxidant imbalance in asthma. In asthma, ROS derived from inflammatory cells (such as epithelial cells, macrophages, neutrophils, and eosinophils) and environmental factors. These inflammatory cells generate amounts of ROS through mitochondrial respiration, NADPH oxidase, and a xanthine oxidase system. The antioxidant enzyme (SOD, CAT, and GPX) activity and the nonenzymatic antioxidants (such as vitamin C, vitamin E, and urate) are reduced in asthma lung. The imbalance regulates various inflammatory factor releases of inflammatory cells, activating inflammatory response and promoting the development and progression of asthma. ROS: reactive oxygen species; NADPH: nicotinamide adenine dinucleotide phosphate; SOD: superoxide dismutase; CAT: catalase; GPX: glutathione peroxidase.

Oxidative stress is defined as the imbalance between ROS production and the endogenous antioxidant defense system, leading to cellular damage [20]. The presence of asthma tends to increase ROS production, and the antioxidant system in the lungs is insufficient to mitigate it. Therefore, asthma can lead to the body under oxidative stress, which exasperates airway hyperresponsiveness and airway inflammation (Figure 1). ROS and reactive nitrogen species (RNS) play vital roles in regulating oxidative stress. Meanwhile, ROS is involved in the onset of inflammatory responses by impacting cell-signaling proteins, such as NF-*Κ*b, TLR, MAPKs, and Keap1-Nrf2-ARE [21–23].

### 2.1. Source of ROS and RNS

ROS is primarily composed of superoxide anion (O2^·^-), hydroxyl radical (^·^OH), and hydrogen peroxide (H_2_O_2_) [24]. Both endogenous and exogenous sources can produce ROS. Endogenous ROS are composed of mitochondrial respiration, NADPH, and a xanthine oxidase system [5]. Asthma is characterized by chronic inflammatory responses in the airways, in which multiple inflammatory cells are recruited and activated, such as macrophages, neutrophils, and eosinophils. These inflammatory cells and epithelial cells generate large amounts of ROS [5]. O2^·^- is formed by the process of reduction of molecular oxygen mediated by an endogenous source and which is broken down by the superoxide dismutase (SOD) into H_2_O_2_. The Fenton reaction produces the highly reactive and toxic hydroxyl radical (^·^OH) when H_2_O_2_ reacts with some transition metals (e.g., Fe2+ and Cu+) [25, 26]. Furthermore, neutrophils and eosinophils release cytotoxic granule proteins, such as myeloperoxidase (MPO) and eosinophil peroxidase (EPO), which catalyze the formation of hypochlorous acid (HOCl) from H_2_O_2_ in the presence of chloride (Cl-) [20, 26]. HOCl is a potent oxidant that kills pathogens in the airways [27]. Exogenous ROS is mainly related to environmental factors, such as smoking, ozone, particulates, and ionizing radiation [26]. These environmental triggers produce many ROS and stimulate inflammatory cells to produce large amounts of TNF-*α*, IL-6, and IL-1*β*, resulting in impaired airway epithelium and capillary endothelial barrier function, resulting in lung injury [28].

Nitric oxide (NO), nitric dioxide (NO_2_), nitrous acid (HNO_2_), and dinitrogen tetroxide (N_2_O_4_) are the constituents of RNS [29]. The level of fractional exhaled nitric oxide (FeNO) is well recognized as elevated in asthma. Nitric oxide (NO) is produced at high levels by inducible nitric oxide synthase (iNOS) by the oxidation of L-arginine (L-Arg) to L-citrulline during bacterial/proinflammatory stimuli [30, 31]. In asthma, airway inflammation induces iNOS expression in macrophages, neutrophils, and epithelial cells [31–34]. Furthermore, cigarette smoke contains a high concentration of NO [35], one of the major oxidative stress sources in the respiratory system. Peroxynitrite anion (ONOO−) is a highly reactive oxidant species that can cause lipid oxidation and damage pulmonary epithelial cells when NO and O2^·^- may react together [36, 37].

### 2.2. Antioxidants

Normally, the lungs have a complete antioxidant system divided into enzymatic and nonenzymatic reactions. The main enzymes that resist ROS are superoxide dismutase (SOD), glutathione peroxidase (GPX), glutathione-S-transferase (GST), and catalase (CAT) [24]. Nonenzymatic antioxidants include vitamins (vitamins C and E), beta carotene, glutathione, and polyphenols, among others [26]. Enzymatic antioxidants of the lungs have been found to play crucial roles in the pulmonary antioxidant defenses. SOD is widely expressed in the human lung, the first and most important line of antioxidant enzyme defense systems against ROS and reduces O2^·^- to H_2_O_2_ [38, 39]. CAT and GPX are the enzymes responsible for reducing H_2_O_2_ to water [26, 40]. Nonenzymatic antioxidants exist in the respiratory tract lining fluids [41]. Vitamins C and E play a key role in protecting lipid peroxidation through their ability to reduce radicals [41]. GSH can scavenge ^·^OH, H_2_0_2_, and HOCl by donating its electrons [26, 41]. Nevertheless, it has been reported that the antioxidant enzyme activity is reduced in the asthma lung and the nonenzymatic antioxidants (such as vitamin C, vitamin E, and urate) are decreased in the respiratory tract lining fluids in asthmatics [42–44].

### 2.3. Redox-Sensitive Signaling Pathway

Oxidative stress affects the redox-sensitive signaling pathways and promotes the development of asthma. Low levels of oxidative stress led to the activation of the Keap1-Nrf2-ARE signaling pathway, inducing the expression of genes encoding antioxidant and detoxifying enzymes, such as heme oxygenase 1 (HO-1), SOD, CAT, and GSTs, which can eliminate the excess ROS [26, 45]. However, at higher levels of oxidative stimuli, which activate the NF-*κ*B, TLR, and MAPK signaling pathways [21, 22, 26], TLRs are important components of the innate immune system, which lead to the activation of transcription factors like NF-*κ*B and activator protein-1 (AP-1) through inhibitor of *κ*B (I*κ*B) kinase (IKK) and MAPKs [46, 47]. Consequently, the expression of inflammatory mediators is upregulated, including proinflammatory cytokines as well as prooxidant enzymes (such as NOX and iNOS), which lead to high levels of ROS. Meanwhile, TLRs also promote mitochondria to produce more ROS. Furthermore, some studies have demonstrated that ROS was also essential for TLR recruitment and dimerization, amplifying the TLR response [48–50]. NF-*κ*B is a master redox-sensitive transcription factor that can be stimulated and inhibited by ROS at different stages of the inflammatory response. ROS could promote NF-*κ*B and increase the expression of TNF-*α*, IL-6, and IL-1*β* in the early phases of the inflammatory response, resulting in impaired airway epithelium and capillary endothelial barrier function [51], but they could also inhibit these responses later on, assisting to induce tissue repair [47, 52]. The MAPK/AP-1 pathway plays a crucial role in oxidative stress [53]; AP-1 regulates inflammatory factors such as TNF-*α*, IL-6, and MCP-1ex [54], which activate an inflammatory response.

## 3. The Mechanisms of Oxidative Stress in Asthma

Asthma is a stepwise process characterized by the gradual accumulation of inflammatory and immune events. Airway inflammation persists throughout the disease and lays the basis for airway hyperresponsiveness and remodeling. In asthma, due to the imbalance between excessive ROS generation and reduced antioxidant defense mechanism, oxidative stress is generated. Oxidative stress plays an important role in the development and progression of asthma.

### 3.1. Oxidative Stress in Airway Inflammation

Asthma is mediated by type 2 and non-type 2 airway inflammations [55–57]. Type 2 inflammation is characterized by the release of T2 cytokines and is involved in the activation and migration of eosinophils, such as IL-4, IL-5, and IL-13. IL-5 promotes the synthesis of immunoglobulin E (IgE). Eosinophils then release mediators, including cytokines (such as IL-13 and IL-5), chemokines, and cytotoxic granule proteins (such as eosinophil cationic protein (ECP) and eosinophil peroxides) [58, 59], causing airway damage and the remodeling of the airway, promoting the occurrence and development of asthma. IL-4 induces IgE isotype conversion in B cells and upregulates the high-affinity IgE receptor (Fc*ε*RI) on the mast cell surface. When bound to IgE, it causes the release of inflammatory mediators, such as histamine, serotonin, prostaglandin D2, and trypsin, which increases smooth-muscle contraction and the excessive secretion of mucus [55, 60]. Non-type 2 airway inflammation is mainly implicated in the abnormal immune responses that are largely orchestrated by neutrophils, which lead to severe asthma [61]. The mechanism of airway neutrophilia in severe asthma is not known; IL-17 appears to play an important role in neutrophilic inflammation, which induces the production of chemokines and cytokines (such as G-CSF, GM-CSF, CXCL1, CXCL6, CXCL8, CSF3, IL-6, and IL-8) by bronchial epithelial cells to elicit neutrophilic airway inflammation [62–64].

**Figure 2:**
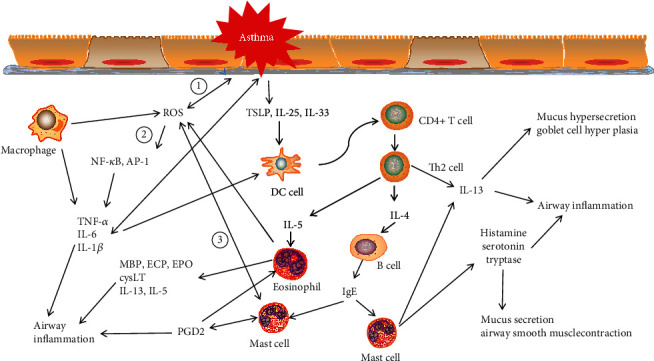
Oxidative stress in asthma airway inflammation. ROS are involved in the progression of asthma airway inflammation through three pathways. ① The increased release of ROS can result in direct oxidative damage to bronchial epithelial cells and cell shedding in asthma, which leads to the activation of epithelial cells and releases cytokines such as IL-25, IL-33, and TSLP. These cytokines promote the production of T2 cytokines from Th2 cells and ILC2s through the activation of dendritic cells (DCs), promoting type 2 inflammation. ② ROS have been implicated in the activation of transcription factors such as NF-kB and AP-1, which promotes the release of IL-6, IL-8, and TNF-*α*, thus activating T2 inflammatory response and resulting in impaired airway epithelium and capillary endothelial barrier function. ③ ROS can stimulate mast cells to release histamine, prostaglandin D2, and other proinflammatory mediators, as well as increase the production of mucus by airway epithelial cells, resulting in airway inflammation. ROS: reactive oxygen species; TSLP: thymic stromal lymphopoietin; NF-kB: nuclear factor kappa-B; AP-1: activator protein-1; MBP: myelin basic protein; ECP: eosinophil cationic protein; EPO: eosinophil peroxidase.

ROS can be generated in asthma by inflammatory cells (such as epithelial cells, macrophages, neutrophils, and eosinophils) and lung epithelial cells [65, 66]. ROS can lead to direct oxidative damage and cell abscission of bronchial epithelial cells in asthma, which activates epithelial cells and releases cytokines such as IL-25, IL-33, and thymic stromal lymphopoietin (TSLP). These cytokines promote the production of T2 cytokines from Th2 cells and ILC2s through the activation of dendritic cells (DCs), promoting type 2 inflammation [67]. Furthermore, ROS has been implicated in activating transcription factors such as NF-*κ*B and AP-1, which promote the release of IL-6, IL-8, and TNF-*α*, thus activating the T2 inflammatory response and resulting in impaired airway epithelium and capillary endothelial barrier function [21]. ROS can stimulate mast cells to release histamine, prostaglandin D2, and other proinflammatory mediators, as well as increase the production of mucus by airway epithelial cells, resulting in airway inflammation [68]. There are prostaglandin D2 receptors on the membrane surfaces of Th2 cells, mast cells, and eosinophils. When it binds to PGD2, it will promote the transmigration of Th2 cells and activate eosinophils to sites of inflammation, releasing IL-4, IL-5, and IL-13 [69] (Figure 2).

### 3.2. Oxidative Stress in Airway Hyperresponsiveness

The fundamental characteristic of the various asthma types is airway hyperresponsiveness (AHR) [70]. T2 cytokines are factors that initiate and accelerate airway hyperresponsiveness in asthma [71]. Among asthma patients, AHR has been related directly to airway smooth muscle contractility changes. Eosinophils can damage airway epithelium and induce ASM contraction and airway hyperresponsiveness by degranulating to release inflammatory mediators and granule proteins, which promote the occurrence and development of asthma. Histamine derived from mast cells, prostaglandin D2, and the cysteinyl leukotrienes are potent spasmogens of airway smooth muscle that cause bronchoconstriction and airway hyperresponsiveness [72, 73]. In addition, mast cells are directly activated by IL-33 and indirectly promote ASM contraction by upregulation of IL-13, enhancing airway responsiveness [74]. In asthma, excessive ROS production can increase lipid peroxidation and the permeability of the alveolar epithelial cells by destroying the cell membrane and promoting the release of proinflammatory cytokines from epithelial cells and alveolar macrophages, which increases the AHR [75–77].

### 3.3. Oxidative Stress in Airway Remodeling

Airway remodeling changes are hallmark pathologic features of asthmatic airway disease, including airway epithelial cell damage, inflammatory cell infiltration, goblet cell proliferation, mucous-gland hyperplasia, airway smooth muscle cell (ASMC) hypertrophy and migration, extracellular matrix (ECM) deposition, airway wall thickening, and increased angiogenesis [70]. Inflammation in asthma drives the pathological structural remodeling of the airways. Various cytokines, chemokines, and growth factors released by inflammatory cells and structural cells in airway tissue are crucial for airway remodeling. In asthma, airway inflammation usually involves Th2 cells, which release IL-4, IL-5, IL-9, and IL-13, and play an important role in the development of airway remodeling. The TH2-mediated inflammatory response can cause specific airway epithelial cell changes, resulting in goblet cell proliferation, epithelial hypertrophy, increased collagen deposition, excessive mucus secretion, and increased airway eosinophils [78–82]. Eosinophils are the main source of the profibrotic cytokine TGF-*β*, which can induce collagen synthesis, fibroblast proliferation, and myofibroblast maturation [83], and play a vital role in tissue remodeling [84]. TGF-*β* is a potent modulator of fibroblast and myofibroblast proliferation and differentiation and can be regulated by the Smad 2/3 and mitogen-activated protein kinase (MAPK) pathways to increase ASM cell proliferation [85, 86]. Furthermore, TGF-*β* has been found to play a role in enhancing the migration of ASM cells to epithelial cells to form new bundles [87]. Also, a recent study shows that autophagy plays a role in airway remodeling and can reduce lung function in asthma patients [88]. In recent years, the activation of autophagy by TGF-*β*1 has been recognized as a biological function of TGF-*β*1 [89, 90]. TGF-*β* can cause the accumulation of autophagosomes and the transformation of microtubule-associated protein-1 light chain 3, as well as increase the mRNA expression levels of autophagy-associated proteins such as Beclin1, Atg5, and Atg7 [90]. Autophagy is crucial for the activation of myofibroblasts and smooth muscle cells [91].

**Figure 3:**
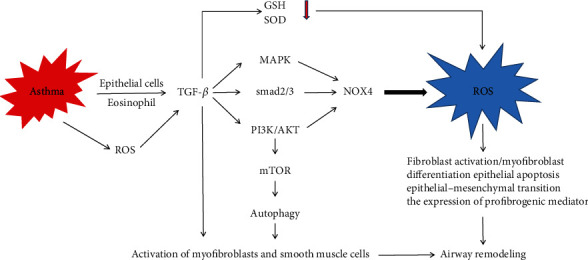
Oxidative stress in asthma airway remodeling. In asthma, eosinophils and airway epithelial cells are the main source of the profibrotic cytokine TGF-*β*, which can stimulate ROS production by activating membrane-related oxidase and inhibiting antioxidant enzymes. TGF-*β* can induce the activity of NAPDH oxidase (NOX) through a variety of signaling pathways, including the SMAD pathway, PI3K pathway, and MAPK pathway, and inhibit the expression of GSH, SOD, and other antioxidant enzymes. Furthermore, TGF-*β* can regulate autophagy by the PI3K pathway, which is crucial for the activation of myofibroblasts and smooth muscle cells. GSH: glutathione; SOD: superoxide dismutase; MAPK: mitogen-activated protein kinase; NOS2: nitric oxide synthase 2; NO: nitric oxide.

ROS stimulates the TGF-*β* excretion in epithelial cells [92]. ROS induces TGF-*β*1 and mediates the TGF-*β*-induced profibrotic effects, including differentiation of airway epithelial cells, subepithelial airway fibrosis, and airway smooth muscle proliferation [93]. TGF-*β* can also activate membrane-related oxidase, which increases ROS production [93]. TGF-*β* can activate NADPH oxidase (NOX) via several signaling pathways, including the Smad pathway, PI3K pathway, and MAPK pathway [93, 94]. TGF-*β* can also induce REDOX imbalance by inhibiting antioxidant enzymes, increasing oxidative stress levels. TGF-*β*1 has been shown in numerous studies to inhibit the expression of GSH, SOD, and other antioxidant enzymes [95–97] (Figure 3).

## 4. PM2.5-Induced Asthma

### 4.1. Source and Components of PM2.5

PM2.5 is one of the atmospheric aerosol pollutants [6], which originates from natural sources (such as forest fires, volcanic eruptions, dust storms, and pollen) and anthropogenic emissions (such as smoking, cooking, vehicle exhaust emissions, and fuel combustion in industrial and agricultural) [98–100]. The components of PM2.5 include heavy metals, sulfuric/nitric/ammonia salts, polycyclic aromatic hydrocarbons, fungi, spores, and viruses [99]. Due to the small particle size (<2.5 *μ*m), large specific surface area, and strong toxin absorption capacity of PM2.5, it is recognized as a major health hazard [100]. PM2.5 can enter the lung tissue through the respiratory tract and deposit in the alveolar regions, causing inflammatory reactions in the lung.

### 4.2. Experimental Findings of PM2.5-Induced Asthma

In recent years, epidemiological studies strongly suggest that an increased risk of asthma exacerbations is associated with elevated exposure to air pollution, especially PM2.5 exposure [6]. PM2.5 has become one of the most significant causes of asthma. Epidemiological analyses have demonstrated an association between short- and long-term exposures to PM2.5 and increased emergency room visits and hospital admissions for asthma exacerbation [17, 101, 102]. A previous research demonstrated that the risk of a child's hospital admission or ED visit was strongly associated with the short-term increase in PM2.5 concentration, with a 4.8% increase for every 10 *μ*g/m^3^ [103]. PM2.5-induced asthma is likely affected by factors such as age, hours of outdoor activity, and local air pollution [104–107]. Asthmatic children are particularly vulnerable to the bad effects of PM2.5 [106]. With the increase in the hours of outdoor activity and the aggravation of air pollution, asthma has increased significantly, and hospital admissions are elevated [106–108].

### 4.3. The Mechanisms of PM2.5-Induced Asthma

Ambient PM2.5 exposure is a major risk factor for type 2 airway inflammation. PM2.5 contains biological components and organic components with REDOX activity which could induce oxidative stress, damage the airway mucosal barrier, and activate type 2 inflammatory responses, such as fungi, spores, viruses, and polycyclic aromatic hydrocarbons [42]. Oxidant stress is believed to be important in PM2.5-induced asthma pathogenesis. Previous studies found that ROS accumulation increased in lung tissues of mice exposed to PM2.5 [109], so did the expression of IL-5 and IL-6 mRNA and TNF-*α* and IL-6 [110].

Exposure to PM2.5 can damage airway epithelial cells, cause lung inflammation and oxidative stress, and induce the release of proinflammatory cytokines and the influx of inflammatory cells into the airway by activating different signaling pathways, including the Nrf2-keap1-ARE signaling pathway, the NF-*κ*B signaling pathway, the MAPK signaling pathway, and the PI3K/Akt signaling pathway [111, 112]. Through the production of antioxidant enzymes and cellular protective proteins, the Nrf2 signaling pathway plays an important role in preventing airway inflammation and oxidative damage caused by PM2.5. Exposure to PM2.5 causes elevated concentrations of calcium ions in airway epithelial cells. Calcium is released from the endoplasmic reticulum and regulates various signal transduction pathways, including the activation and phosphorylation of the MAPK family, which leads to increased gene transcription (NF-*κ*B, AP-1) that promotes the release of IL-6, IL-8, and TNF-*α* [113], thus activating T2 inflammatory response and inducing airway hyperreactivity.

**Figure 4:**
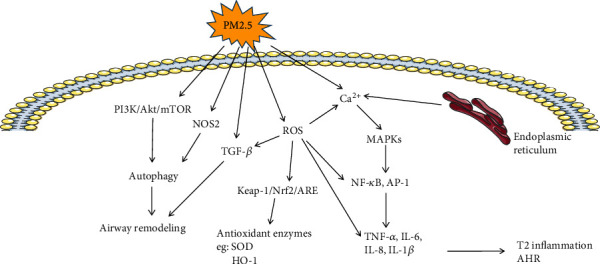
The mechanisms of PM2.5-induced asthma. Exposure to PM2.5 can damage airway epithelial cells, cause lung inflammation and oxidative stress, and induce the release of proinflammatory cytokines (IL-6, IL-8, and TNF-*α*). Meanwhile, exposure to PM2.5 causes airway epithelial cells and the endoplasmic reticulum release calcium ions, which activates and phosphorylates the MAPK family, leading to increased gene transcription (NF-*κ*B, AP-1), promoting the release of IL-6, IL-8, and TNF-*α*, thus activating T2 inflammatory response and inducing airway hyperresponsiveness. In addition, exposure to PM2.5 can induce autophagy through the PI3K/Akt/mTOR signaling pathway and elevate autophagy by affecting the expression of nitric oxide synthase 2 (NOS2) and the production of NO, as well as TGF-*β*1 expression, causing airway remodeling. SOD: superoxide dismutase; HO-1: heme oxygenase 1; AHR: airway hyperresponsiveness.

An animal study found that prolonged exposure to PM2.5 increased TGF-*β*1 expression, smad2/3 phosphorylation levels, and collagen accumulation in the lungs of mice [114]. In another study, it was found that alveolar epithelial cells exposed to PM2.5 induced TGF-*β* expression and changed cell morphology and increased cell contractility [115]. In addition, PM2.5 exposure can induce autophagy through different molecular mechanisms, thus affecting the development of airway remodeling in asthma. A study has shown that PM2.5 promotes autophagy by affecting the expression of nitric oxide synthase 2 (NOS2) and the production of NO [89]. Other studies have shown that PM2.5 can induce autophagy in BEAS-2B cells through the PI3K/Akt/mTOR signaling pathway [90] (Figure 4).

## 5. Treatment of Asthma with Traditional Chinese Medicine

Asthma is the most common chronic disease worldwide. Although long (short)-acting *β*-agonists and inhaled corticosteroids (ICS) are effective for asthma, there are currently no effective treatments for the disease, and many patients continue to suffer from the disease exacerbation. However, *β*-agonists and steroids have significant adverse effects, especially in long-acting *β*-agonists. Monotherapy significantly increases the risk of cardiovascular disease [116]. Recently, the application and effectiveness of using small-molecule compounds in traditional Chinese medicine for asthma have been proven by many researchers due to their distinct pharmaceutical value and fewer side effects [117]. Many studies are researching potential antiasthma drugs that have been used in traditional Chinese medicine, including polyphenols, flavonoids, alkaloids, terpenoids, emodin, cryptotanshinone, and catalpol [118] (Table 1). Nearly all of these medicines have the effect of antioxidants. The mechanisms of traditional Chinese medicine are mediated by modulation on multiple redox-sensitive signaling pathways. One study found that zingerone, which belongs to polyphenols, reduces inflammation in asthma by acting on the AMPK/Nrf2/HO-1 signaling pathway [119]. Icariin, a flavonoid, can reverse immune imbalance in asthma by downregulating GATA-3 and NF-*κ*B, while upregulating T-bet [120]. Other research found that matrine (which belongs to alkaloids) can block asthma progression by downregulating IL-4/IL-13/STAT-6 and NF-*κ*B [121, 122]. In a word, traditional Chinese medicine can regulate the inflammatory response by acting on the redox-sensitive signaling pathways, thereby regulating the progression of asthma, which may represent a new option for asthma treatment.

## 6. Conclusion

PM2.5 exposure is correlated with asthma and is closely related to asthma severity. This review elaborated on the molecular mechanism of PM2.5-induced asthma from three aspects: airway inflammation, airway hyperresponsiveness, and airway remodeling. It is worth noting that almost all of these mechanisms rely on oxidative stress balance. Oxidative stress plays a central role in the pathogenesis of PM2.5-mediated asthma. Therefore, oxidative stress should be considered in future asthma treatment. Moreover, traditional Chinese medicine, which has the effect of antioxidants, may represent a new option for asthma treatment.

## Figures and Tables

**Table 1:** The effects of traditional Chinese medicine on asthma.

Categories	Monomers	Animal species	Related signaling pathway	References
Polyphenols	Curcumin	BALB/c mice	MAPK/NF-*κ*B↓	[123]
	Zingerone	BALB/c mice	AMPK/Nrf2/HO-1↑	[119]
	Resveratrol	Sprague Dawley rats	HMGB1/TLR4/NF-*κ*B↓	[124]
	Resveratrol	Sprague Dawley rats	Keap-1/Nrf2↑	[125]
	Luteolin	BALB/c mice	PI3K/Akt/mTOR↑	[126]
	Quercetin	BALB/c mice	GATA-3↓ and T-bet↑	[127]

Flavonoids	Diosmetin	BALB/c mice	MMP-9, TGF-*β*1, VEGF↓	[128]
	Galangin	BALB/c mice	TGF-*β*1-ROS-MAPK↓	[129]
	Icariin	Sprague Dawley rats	GATA-3↓ and T-bet↑ NF-*κ*B↓	[120]

Alkaloids	Ginsenoside Rh1	BALB/c mice	Eotaxin, IL-4, IL-5, IL-13, and IL-33↓ IL-12 and IFN-*γ*↑	[130]
	Ligustrazine	C57BL/6J mice	GATA-3↓ and T-bet↑ ROR*γ*t↓	[131]
	Matrine	BALB/c mice	NF-*κ*B↓	[122]

Terpenoids	Andrographolide	BALB/c mice	NF-*κ*B↓	[132]

Anthraquinones	Emodin	Sprague Dawley rats	NF-*κ*B↓	[133]

Diterpene quinones	Cryptotanshinone	BALB/c mice	p38 MAPK and NF-*κ*B↓	[134]

Iridoids	Catalpol	BALB/c mice	TGF-*β*1 and EGF↓	[135]

## Data Availability

All data used to support the findings of this study are included within the article.
